# Acute interstitial nephritis after sequential ipilumumab - nivolumab therapy of metastatic melanoma

**DOI:** 10.1186/s40425-017-0261-2

**Published:** 2017-07-18

**Authors:** Lea Bottlaender, Anne-Laure Breton, Louis de Laforcade, Frederique Dijoud, Luc Thomas, Stephane Dalle

**Affiliations:** 1 Service de Dermatologie, ImmuCare, Hospices Civils de Lyon, Centre Hospitalier Lyon-Sud, Pierre-Bénite cedex, France; 2 Université Claude Bernard Lyon 1, Lyon, France; 3 Centre de Recherche en Cancérologie de Lyon, Lyon, France; 4 Service de Néphrologie, Hospices Civils de Lyon, Centre Hospitalier Lyon-Sud, Pierre-Bénite cedex, France; 5 Service d’Anatomie et de Cytologie pathologique, Hospices Civils de Lyon, Groupement Hospitalier Est, Bron cedex, France

**Keywords:** Pd-1, Melanoma, Acute kidney injury, Acute interstitial immune nephritis, Immunotherapy

## Abstract

**Background:**

The anti-Programmed Death receptor 1 (anti-PD-1) antibodies nivolumab and pembrolizumab are new treatments in metastatic melanoma. Immunotherapies are best known to be responsible for thrombotic microangiopathy. However, immune interstitial nephritis has been described in a patient treated by nivolumab and ipilimumab concomitantly, and three cases of granulomatous interstitial nephritis have been reported with ipilimumab monotherapy. We report herein a case of acute interstitial immune nephritis in a patient treated with nivolumab after ipilimumab for pulmonary metastatic melanoma.

**Case presentation:**

Interstitial nephritis was diagnosed after acute kidney injury following three cycles and was confirmed by kidney biopsy. Kidney injury responded rapidly to prednisolone, which was then gradually reduced. As a follow-up computed tomography scan indicated mixed response, with minimal size progression of a pulmonary nodule, but a significant reduction in the size of the other nodules, nivolumab was reintroduced after renal function improvement. Low-dose corticosteroids were first maintained during nivolumab treatment and subsequently discontinued. Only one month after prednisolone discontinuation, creatinine levels increased. A second kidney biopsy confirmed relapse of acute interstitial nephritis.

**Conclusions:**

To our knowledge, this is the first case of nivolumab-induced acute interstitial immune nephritis. This case highlights that anti-PD-1 immunotherapy may be continued when renal function is adequate, and this requires close interaction between dermatologists and nephrologists. This adverse effect should be made known to prescribers as nivolumab is associated with significant improvement of survival in metastatic melanoma and may be used in many different types of cancer.

## Background

The anti-Programmed Death receptor 1 (PD-1) antibodies nivolumab and pembrolizumab are new therapies in metastatic melanoma [1, 2] and immunotherapy aimed at this target is expanding to other cancers. Immunotherapies are best known to be responsible for thrombotic microangiopathy. However, immune interstitial nephritis has been described in a patient treated by nivolumab and ipilimumab concomitantly [3], and three cases of granulomatous interstitial nephritis have been reported with ipilimumab monotherapy [4, 5].

## Case report

A 76–year-old woman was diagnosed in October 2012 with an anal canal non-mutated BRAF melanoma (Mucosal Lentiginous Melanoma in vertical growth phase, Breslow thickness 20 mm, presence of ulceration, pT4bN0M0, stage IIC). Sentinel lymph node was negative and wide local excision of in situ melanoma was confirmed to have safe margin. She also had a history of high blood pressure that was controlled by olmesartan, a right invasive ductal carcinoma (breast cancer, Scarff, Bloom and Richardson score III, pT1cN0M0) treated by surgery, radiotherapy and anastrozole in June 2013, asthma, and asymptomatic distal pulmonary embolism. Multiple metastatic pulmonary nodules were discovered in January 2015. Melanoma relapse was proven by histological examination of a lung nodule biopsy. From January 19, 2015 to March 23, 2015 she received 4 intravenous cycles of ipilimumab (3 mg/kg) for first-line treatment. She presented only grade 1 diarrhea; no additional immune adverse event was observed. Ipilimumab response was evaluated at 16 weeks and disease progression was found (according to RECIST criteria). Second-line treatment with nivolumab (3 mg/kg) was initiated on May 18, 2015 (8 weeks after ipilimumab discontinuation).

She presented acute kidney injury after three cycles of nivolumab: creatinine was 69 μmol/L before nivolumab initiation, 75 μmol/L before the second cycle, 94 μmol/L before the third cycle, and 142 μmol/L before the fourth cycle. Immunotherapy was discontinued and a non-contrast computed tomography (CT) scan confirmed the absence of any obstacle in the urinary tract. She did not receive any other drug that could explain the increased creatinine level. Stage 2 acute kidney injury was estimated according to KDIGO criteria. Urinalysis found subnormal microscopic glomerular hematuria (18/mm^3^), and leukocyturia (34/mm^3^) mainly composed of neutrophils; protein excretion, infection, eosinophils, lymphocytes, and crystal deposits were not found. Moreover, serum protein electrophoresis was normal. The patient was not taking nephrotoxic drugs and was otherwise asymptomatic. Despite an adequate fluid intake over 3 days, renal failure persisted and therefore renal biopsy was performed.

**Fig. 1:**
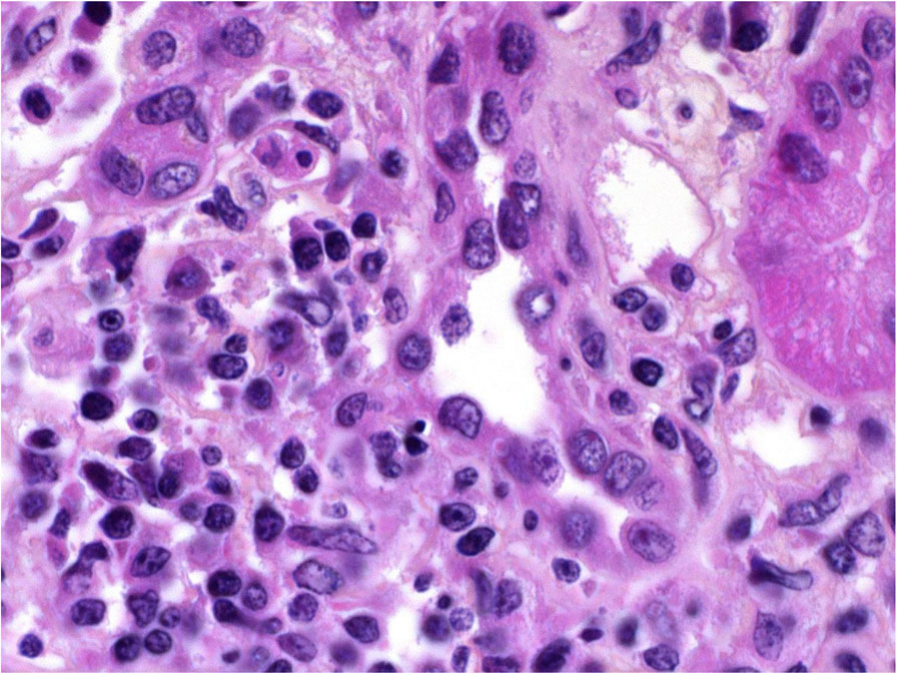
Kidney pathology specimen, HES ×1000 (hematoxylin, eosin, saffron). Interstitial infiltrate with plasma cells associated with patchy tubular necrosis

Morphological examination of the kidney (Fig. 1) found interstitial edema with dense and nodular inflammatory infiltrates with tubulitis and patchy tubular necrosis. The inflammatory cells were predominantly mononuclear, with focally numerous plasma cells and some eosinophils without any granular or giant cell. Neither glomerular nor vascular lesion was found. No deposit was observed by indirect immunofluorescence. Nivolumab-induced acute immune interstitial nephritis was diagnosed.

**Fig. 2:**
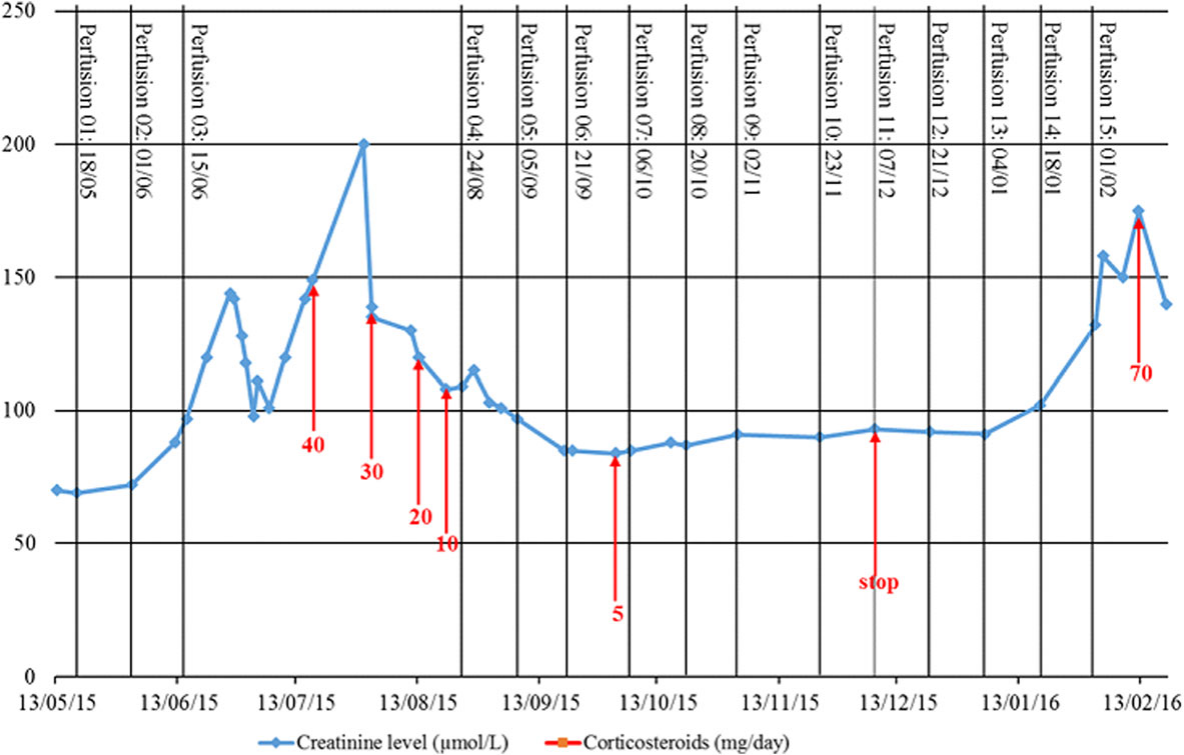
Renal function and corticosteroids. Acute kidney injury appeared after the third cycle of nivolumab. As rehydration was not sufficient, corticosteroids at 0.5 mg/kg/day were initiated. The evolution was marked by functional acute renal injury. At the end of July 2015 creatinine reached 200 μmol/L, olmesartan was discontinued, and with rehydration renal function returned to baseline. Creatinine levels improved and corticosteroids were progressively reduced and then discontinued. Nivolumab was restarted without initial kidney relapse. A new acute renal injury occurred less than two months after discontinuation of corticosteroids

This renal injury was then treated by oral prednisolone at a daily dose of 0.5 mg/kg (40 mg). Renal function rapidly improved although creatinine remained higher than before initiation of nivolumab (Fig. 2) and glomerular filtration rate was estimated to be 42 mL/min/1.73m^2^ (Chronic Kidney Disease - Epidemiology Collaboration method) at the end of August. The dose of corticosteroids was gradually reduced to 10 mg daily by the end of August 2015 (Fig. 2).

A follow-up CT scan performed in August 2015 found a mixed response with minimal size progression of a pulmonary nodule, but a significant reduction in the size of the other nodules. The patient had partial response according to the RECIST criteria. The continuation of nivolumab at the same dose was decided during the multidisciplinary meeting. The fourth cycle was administrated at the end of August 2015 while the patient was still receiving 10 mg of prednisone daily. This infusion did not cause kidney failure relapse. Creatinine was 84 μmol/L at the end of September 2015. Prednisone was then reduced to 5 mg daily and discontinued on December 6, 2015. At this time, pulmonary nodules were stable or in regression, while secondary brain lesions appeared and were managed by stereotaxic radiation therapy.

After discontinuation of corticosteroids, creatinine increased gradually during January 2016 and was 158 μmol/L on February 3, 2016 (two days after the 15th cycle). The patient was hospitalized for acute kidney injury. Urinalysis and blood test findings were similar to those during the first episode of acute kidney injury. Rehydration was not effective and a second renal biopsy was performed to document relapse. This confirmed relapse of immune interstitial nephritis as evidenced by a dense inflammatory interstitial infiltrate with many plasma cell, eosinophils, and a multinucleated giant cell. There were severe tubular lesions. Immunofluorescence and BK virus (in situ hybridization) were negative. There was no evidence for another cause of acute kidney injury. Prednisolone was then initiated at 1 mg/kg daily, and acute interstitial nephritis improved rapidly. Nivolumab was discontinued.

## Discussion and conclusion

Nivolumab is a fully human IgG4 monoclonal antibody raised against PD-1. This cell surface receptor negatively controls T cell proliferation and functions, and is observed on activated T lymphocytes and T cells with chronic stimulation [6]. Studies have, however, found that PD-1-deficient mice develop lupus-like glomerulonephritis, with IgG3 and C3 deposits in glomeruli upon histological examination [6].

Drug-induced acute interstitial nephritis has been more often described with non-steroidal anti-inflammatory drugs, beta-lactams, rifampin, allopurinol, etc., and represents an immune reaction with an underlying cellular or humoral mechanism [7, 8]. On the one hand, immune interstitial nephritis could be explained by specific antibodies against drugs which may also recognize the tubular membrane filter or complement deposited in the kidney interstitial space [9]. On the other hand, by stimulating T lymphocytes, nivolumab could induce acute interstitial nephritis. Blocking PD-1 may provoke cytotoxic T cell effects in the periphery, not only against tumor cells but also against normal tissues. It is of note for the present case that there is upregulation of Programmed death-ligand 1 in renal tissue which will bind to PD-1 expressed by T cells and signal to prevent proliferation and tissue damage. Therefore, when PD-1 signaling is interrupted by anti PD-1 (for instance nivolumab), T cells will proliferate and cause kidney injury [10, 11].

Immunotherapies are best known to be responsible for thrombotic microangiopathy, and this adverse effect is mostly described with anti-vascular endothelial growth factor agents, for instance bevacizumab, aflibercept, etc. [12, 13]. Immune interstitial nephritis has, however, been described in patients treated by nivolumab (0.3 mg/kg or 1 mg/kg) and ipilimumab (3 mg/kg) concomitantly [3, 10]. Authors have suggested that cytotoxic lymphocyte antigen-4 (CTLA-4) modulated the interaction between antigen-presenting cells and T cells in secondary lymphoid organs [10]. Treatment with anti-PD-1 (nivolumab) and anti-CTLA4 (ipilimumab) could potentiate antigen recognition and T cell proliferation at lymph nodes, that may provoke cytotoxic T cell effects in tissues and be responsible for immune acute nephritis [10]. Furthermore, three cases of granulomatous interstitial nephritis have been reported with ipilimumab monotherapy [4, 5]. To our knowledge, no case of immune interstitial nephritis has been reported with nivolumab monotherapy in metastatic melanoma. With regards to the case presented here, it is important to consider that she received ipilimumab just before nivolumab which was initiated 8 weeks after the last ipilimumab infusion. Due to the ipilimumab elimination half-life, which is 15.4 days, we cannot formerly exclude an overlap between ipilimumab and nivolumab that may have increased the risk of acute interstitial nephritis.

Removal of the offending agent is usually the mainstay treatment of immune interstitial nephritis [9], otherwise this acute interstitial nephritis can lead to a chronic interstitial nephritis. In the present case, mixed tumor response observed on the follow-up CT scan encouraged us to continue treatment with nivolumab when renal function recovered. However, nephropathy relapse occurred. The medical decision was then to permanently discontinue the anti-PD1 therapy. We could also consider that nivolumab benefits would have been persistent without re-introduction of the drug and suggest a wait-and-see management. The management of such a situation is still controversial.

This adverse event should be known to prescribers as anti-PD-1/PD-L1 antibodies may be used in many different types of cancer including, for instance, squamous cell lung cancer, non-small cell lung cancer, renal cell carcinoma, head and neck cancer, Hodgkin’s disease, and bladder cancer [14, 15]. The anti-PD-1 immunotherapy may be continued when the renal function is adequate which requires close interaction between cancer immunotherapists and nephrologists. Moreover, the ipilimumab and nivolumab combination have now be approved for treatment of metastatic and is associated with more frequent and severe adverse events [16, 17]. Immune interstitial nephritis, as many immune-related adverse events occurring with immune check point inhibitors, may be managed by the rapid introduction of immunosuppressive agents.

## Abbreviations

CTLA-4: Cytotoxic lymphocyte antigen-4
CT-scan: Computed Tomography Scan
PD-1: Programmed Death receptor 1
